# Determinants of self-rated health among an older Tibetan population in a Chinese plateau area: analysis based on the conceptual framework for determinants of health

**DOI:** 10.1186/s12889-021-10359-x

**Published:** 2021-03-11

**Authors:** Yajie Li, Qucuo Nima, Bin Yu, Xiong Xiao, Peibin Zeng, Deji Suolang, Ruifeng He, Zhuoga Ciren, Pingcuo Wangqing, Ciren Laba, Yangzong Silang, Ling Song, Yixi Kangzhu, Jingzhong Li

**Affiliations:** 1 Tibet Center for Disease Control and Prevention, Lhasa, China; 2 West China Second University Hospital of Sichuan University and Key Laboratory of Birth Defects and Related Diseases of Women and Children (Sichuan University), Ministry of Education, Chengdu, China; 3 West China School of Public Health and West China Fourth Hospital, Sichuan University, Chengdu, China

**Keywords:** Self-rated health, Social determinants of health, Plateau area, Older people

## Abstract

**Background:**

Self-rated health (SRH) has been frequently used in population health surveys. However, most of these studies only focus on specific factors that might directly affect SRH, so only partial or confounding information about the determinants of SRH is potentially obtained. Conducted in an older Tibetan population in a Chinese plateau area, the aim of our study is to assess interrelationships between various factors affecting SRH based on the conceptual framework for determinants of health.

**Methods:**

Between May 2018 and September 2019, 2707 Tibetans aged 50 years or older were recruited as part of the China Multi-Ethnic Cohort Study (CMEC) from the Chengguan District of Lhasa city in Tibet. The information included SRH and variables based on the conceptual framework for determinants of health (i.e.*,* socioeconomic status, health behaviors, physical health, mental health, and chronic diseases). Structural equation modeling (SEM) was used to estimate the direct and indirect effects of multiple factors in the conceptual framework.

**Results:**

Among all participants, 5.54% rated their health excellent, 51.16% very good, 33.58% good, 9.12% fairly poor and 0.59% poor. Physical health (*β* = − 0.23, *P* <  0.001), health behaviors (*β* = − 0.44, *P* <  0.001), socioeconomic status (*β* = − 0.29, *P* <  0.001), chronic diseases (*β* = − 0.32, *P* <  0.001) and gender (*β* = 0.19, *P* <  0.001) were directly associated with SRH. Socioeconomic status, physical health and gender affected SRH both directly and indirectly. In addition, there are potential complete mediator effects in which age and mental health affect SRH through mediators, such as physical health, health behaviors and chronic diseases.

**Conclusions:**

The findings suggested that interventions targeting behavioral changes, health and chronic disease management should be attached to improve SRH among older populations in plateau areas without ignoring gender and socioeconomic disparities.

## Background

Self-rated health (SRH), which is generally evaluated based on a simple question of “How do you think of your present health?” [1], refers to an individual’s expectation and evaluation of health status based on their subjective feelings and objective health information from physical, psychological and social adaptation [2]. Since the 1960s, SRH has been widely used to measure health inequalities in public health research for easy data collection and low costs [3, 4]. As independent predictors for certain health outcomes (e.g.*,* all-cause mortality, disease-specific mortality, morbidity and health service utilization) [5–8], SRH involves a large scope of health issues related to social, economic, behavioral, and psychological factors [9, 10]. Currently, SRH is increasingly used to evaluate and monitor individual and population health status, similar to the predisease warning sign.

Previous studies have found that physical activity, drinking, tobacco use, chronic diseases, functional status, and psychosocial symptoms exhibited a significant association with SRH [11–13]. Although a large number of studies in Western countries have focused on SRH in recent years, most studies in this field only focus on specific factors affecting SRH and rarely consider that many of these factors have complex interrelationships that direct or indirect affect SRH [9, 14]. To completely understand SRH, an in-depth exploration of the structure of SRH is required to clarify which factors are not only directly but also indirectly related to it. However, only a few studies have investigated the direct and indirect associations of health-related factors, chronic diseases, or psychological health with SRH [15, 16]. There is still limited understanding of the factors that individuals refer to in assessing their health and how they act and interact to influence the final assessment. Investigations need to concomitantly assess the factors associated with SRH. Currently, the conceptual framework for determinants of health has been developed to represent complex networks of casual pathways through which social factors interact with an individual’s health risk and protective factors throughout the life course. As a comprehensive model comprising multidimensional variables, the conceptual framework for determinants of health might be helpful to estimate the factors associated with SRH with the presentation of direct and indirect relation paths.

In China, SRH was included in the national population survey data for the first time in 2003 with approximately 94.2% of the population rating their health as ‘very good’ ‘good’ or ‘fair’ [17]. China, which has the largest number of older people, has entered a period of population aging since 2000. By the end of 2017, the number of older people aged 60 years and older reached 240.90 million, accounting for 17.3% of the total population [18]. The concept of healthy aging has become a global health strategy in response to population aging [19]. As physical function declines with age, older people are more likely to suffer from health problems (e.g.*,* chronic diseases, depression, and disability) compared with their younger counterparts and are facing serious health care challenges [20–22]. Therefore, there is a need to better understand the most significant health aspects in older people and how SRH is structured among them. However, there have been limited studies about the influential factors of SRH among the Chinese aging population.

The Tibetan population, the majority of whom live in the Chinese plateau area, includes a large number of older people suffering from more adverse health outcomes and diseases, such as hypertension [23] and obesity [24], compared with the non-Tibetan population. As a study conducted in China found that the overall health status of the residents in the Tibet Autonomous Region is worse than that of other provinces in China [25]. The SRH of this vulnerable population is of concern. In addition, some special environmental factors, genetic factors, lifestyle, and health outcomes [26] may affect SRH. In such a scenario, it is crucial to better identify the multiple dimensions contributing to SRH.

To better understand the influencing factors of SRH in older Tibetan people in a Chinese plateau area, we aimed to reconstruct the system of relations connecting the different factors that affect SRH based on the conceptual framework for determinants of health. Most of the studies in this field only involve specific factors that might directly affect SRH; thus, only partial or confounded information about the determinants of elderly health can be obtained. Investigations in our study assess interrelationships between various factors affecting SRH and the direct or indirect effects of each factor on SRH within the conceptual framework. These evidence-based results might be helpful to implement appropriate public health policies and programs to improve the overall health of the population.

## Materials and methods

### Study design

This cross-sectional study is part of and based on the baseline of the China Multi-Ethnic Cohort Study (CMEC), a community population-based study conducted between May 2018 and September 2019 in five provinces (Sichuan, Chongqing, Yunnan, Guizhou, and Tibet) of southwestern China. Detailed information about the CMEC study rationale, design, survey methods, and participant characteristics was reported previously [27]. The data used in the study are from the area of Chengguan District, Lhasa city, the capital of the Tibetan autonomous region in China. Lhasa is located in the middle of the Tibetan Plateau with an average elevation of 3650 m, making it one of the highest cities in the world. In 2017, Chengguan District had a population of 215,804 inhabitants living on 12 streets. In addition, there were 31 ethnic groups (e.g.*,* Tibetan nationality, Han nationality, and Hui nationality), among which the Tibetan population accounted for 87%.

### Sampling procedure

A multistage cluster sampling method was used to recruit the study participants from the community-based population. First, we chose the Chengguan district of Lhasa in Tibet, which had the largest population density and convenient transportation, as the project site to facilitate the follow-up and management of the cohort population. Second, five out of the 12 streets were selected randomly, namely, Caigongtang Street, Niangre Street, Duodi Street, Najin Street, and Jinzhu West Road Street. Finally, all participants who met the inclusion criteria in the selected streets were invited to participate. The inclusion criteria were as follows: 1) Tibetan nationality; 2) living in the region for three generations or more; and 3) aged 30 or older. Those study participants who were not able to provide a unique national identification card, could not communicate independently and refused to comply with the requirements of the study were excluded from the study.

### Data collection

Data were collected via electronic questionnaires and medical examinations. The questionnaire was developed from the Kadoorie Study of Chronic Disease in China [28], chronic diseases and nutrition surveillance among Chinese adults [29], and other relevant literature [30] based on the study objectives. The information was collected through face-to-face interviews by 35 well-trained interviewers recruited from Tibetan university students with medical backgrounds.

Of 7441 participants, 4550 were aged under 50 years old, and 184 were excluded due to missing records on height, weight, or other important research variables. Finally, a total of 2707 participants were included and analyzed in this study.

### Measurement

#### Self-rated health

The question used for self-rated health was “How do you think of your present health?” The respondents were asked to provide an overall assessment of their current health status. The response options were (a) very good (5-score), (b) fairly good (4-score), (c) average (3-score), (d) fairly poor (2-score), and (e) poor (1-score).

#### Demographics

Demographics included age (classified as 50–54, 55–59, and 60+), gender (male vs. female), and marital status (cohabited [married or not] vs. did not cohabit [(Separated/divorced/widowed and never married)]).

#### Socioeconomic status

Socioeconomic status included educational attainment, employment, household annual income, registered permanent residence, and medical insurance. Educational attainment was classified into three groups: 1 (middle school and above), 2 (elementary school) and 3 (no formal education). Employment was classified into two groups: 1 (employed) and 2 (unemployed). Household annual income was classified into four groups: 1 (≥ 60,000 CNY), 2 (20000–59,999 CNY), 3 (12000–19,999 CNY) and 4 (≤ 12,000 CNY). Registered permanent residence was classified into three groups: 1 (unified household), 2 (urban) and 3 (rural). Medical insurance was coded according to the level of security covered by the government-funded social health insurance program: 1, Medical Insurance for Urban Employees (MIUE); 2, Medical Insurance for Urban Residents (MIUR); 3, New Rural Cooperative Medical Scheme (NCMS); 4, Medical Insurance for Rural Residents (MIRR); and 5, no medical insurance coverage.

#### Health behaviors

Health behaviors included smoking, alcohol consumption, quality of nighttime sleep, sleep duration, and physical activity. Smoking was assessed by an item that asked, “Do you smoke?”. There are three response options, namely, never (smoked less than 100 cigarettes during their life), ever (not currently smoking), and current (smoking at least one cigarette or less than one cigarette a day) [31]. Alcohol consumption was assessed with the frequency choice of “none”, “once a week or less”, or “more than once a week”. Quality of nighttime sleep score was generated by collating responses from the following four items: (a) “It took more than half an hour to fall asleep, at least three days a week” (b) “I woke up very early and had been difficult to fall asleep again, at least three days a week” (c) “I took sleeping pills, at least one day a week” and (d) “I had difficulty concentrating when working, eating or talking in the daytime because of poor sleep last night, at least three days a week.” Each of the four items included no or yes response options, which were given a corresponding value of zero or one, respectively. Responses from each item were summed to calculate the overall quality of the nighttime sleep score. A higher total score indicates poor quality of nighttime sleep. Sleep duration was assessed by an item that asked, “On average, how many hours do you usually sleep per night last month?”. According to previous studies [32], 7–8 h/night was considered adequate sleep duration for older people. In addition, short sleep and long sleep duration were associated with poor SRH, with short sleep seeming to affect SRH more seriously [33, 34]. Thus, participants’ responses were scored as 1 (7–8 h/night), 2 (> 9 h/night), and 3 (< 7 h/night). Physical activity considered participants’ job-related physical activity, transportation physical activity, leisure-time physical activity, and housework and was divided into two groups based on the median metabolic equivalent for task (MET).

#### Mental health

Mental health was assessed using two variables: depression and anxiety. Depression was measured as a continuous variable using the Patient Health Questionnaire-2 (PHQ-2) [35], namely, (a) “Little interest or pleasure in doing things” and (b) “Feeling down, depressed, or hopeless”. The PHQ-2, a short version of the PHQ-9, is composed of the first two items of the PHQ-9 [36]. The internal reliability of the PHQ-2 has been reported at Cronbach’s α = 0.76 in older Chinese rural people [37]. The reliability of vitality in this study (Cronbach’s α = 0.76) was similar to that of this report. Anxiety was measured as a continuous variable using the 2-item Generalized Anxiety Disorder Questionnaire (GAD-2) [38], namely, (a) “Not being able to stop or control worrying” and (b) “Feeling nervous, anxious, or on edge”. The GAD-2 is an abridged version of the tool that is composed of the first two items of the GAD-7 [39]. There is evidence demonstrating good reliability and validity of the PHQ-2 (Cronbach’s α = 0.82) [40]. The reliability of vitality in our study (Cronbach’s α = 0.72) was comparatively lower compared with that in these reports.

The score of each item ranges from 0 (not at all) to 3 (nearly every day). The total score is obtained by the simple addition of item scores and ranges between 0 and 6. A higher total score indicates more mental health problems.

#### Physical health

Mobility, pain, activities of daily living (ADL), and self-care were used as measures for physical health. Mobility was based on the question “Is it difficult for you to get around?”. Pain was based on the question “Do you have any pain or discomfort today?”. ADL was based on the question “Do you have any difficulties with your daily activities today?” Self-care was based on the question “Do you have any trouble taking care of yourself today?”. For each item, the options were (a) “none”, (b) “a little bit”, (c) “moderate”, (d) “serious”, and (e) “very serious”. The score of each item ranges from 0 (none) to 5 (very serious).

#### Chronic diseases

Four chronic diseases were included in the study: obesity, hypertension, hyperlipidemia, and diabetes. Height and weight were collected during the medical examination, and body mass index (BMI) was calculated as weight in kilograms per height in squared meters. Obesity was defined as having a BMI ≥ 28.00 according to China’s BMI criterion [41]. For hypertension, hyperlipidemia, and diabetes, participants were asked whether they had been diagnosed with these diseases by a doctor.

### Statistical analysis

The conceptual framework for determinants of health [42], which was developed by the commission on social determinants in 2008, consists of the following three key components: (a) the socioeconomic and political environment; (b) the individual socioeconomic status, including the status and degree of social stratification by gender and race; and (c) the intermediate determinants of health, including the differential material environment, social psychology, behavioral factors and health service status of different populations.

According to the conceptual framework for determinants of health, we built a hypothesized model with measurement SRH as the dependent variable. To concomitantly explore factors associated with SRH yet retain a parsimonious model, we delimited the study by restricting the explanatory variables to the following: socioeconomic status (educational attainment, employment, household annual income, medical insurance, and registered permanent residence), health behaviors (smoking, alcohol consumption, sleep duration, physical activity and quality of nighttime sleep), physical health (anxiety and depression)**,** mental health (mobility, pain, take care of myself, and activities of daily living), and chronic diseases (hypertension, diabetes, hyperlipidemia and obesity). The Kruskal-Wallis test was used to compare the differences in SRH between groups. The total scores of anxiety and depression were not normally distributed, and the differences in these variables and SRH were tested using the Mann–Whitney U-test. Structural equation modeling (SEM) was used to estimate the model fitting degree of the data, and the direct and indirect effects of multiple factors in the hypothesis model were analyzed. Demographics (age and gender) are inevitably exogenous and can only be affected by other factors rather than by themselves.

For all factors included in the SEM, higher scores indicate older age, male gender, lower educational attainment, unemployment, lower household annual income, lower entitlement insurance schemes, rural registered permanent residence, having more physical health problems, never smoking or drinking alcohol, poor quality of nighttime sleep, having more mental health problems, and having been diagnosed with chronic diseases.

Multiple indicators were used to evaluate the fit of the model, namely, relative *χ*^2^ (CMIN/DF) and baseline comparison fit indices of NFI, RFI, IFI, TLI, CFI, RMR and RMSEA [15]*.* All analyses were performed using SPSS (IBM, Armonk, NY, USA) and AMOS 21.0 statistical software (IBM, Armonk, NY, USA). The association was considered to be statistically significant if the 2-sided *P*-value was less than 0.05.

## Results

### Characteristics of participants

A total of 2707 participants were analyzed in this study, and 60.02% of them were female. The mean age of the participants was 58.8 ± 6.8 years, ranging from 50 to 84 years old. The majority (87.44%) of participants cohabited, and 1969 (72.24%) were unemployed. Greater than half of the participants (62.87%) had received no formal education; only 7.24% had completed middle school or above education. Their household annual income was distributed evenly across the four ranges. Participants were less likely to report smoking (15.11% for the current smoker and 6.83% for ever smoker) and drinking (17.33% drank less than once a week and 8.79% drank more than once a week). The median level of physical activity was 18.9 MET/day. Most (65.87%) participants reported an adequate sleep duration between 7 and 8 h. The self-reported prevalence rates of chronic diseases ranged from high to low followed by hypertension (31.29%), hyperlipidemia (25.60%), obesity (19.95%) and diabetes (3.58%). The median scores of nighttime sleep quality, anxiety, and depression were 0 (0,1), 0 (0,1), and 0 (0,1), respectively (Table 1).

**Table 1:** Characteristics of participants and self-rated health (*n* = 2707)

Characteristics of participants	Full sample	Self-rated health status					*P-*value
		Poor	Fair	Good	Very good	Excellent	
*Demographics*							
Gender ^a^							< 0.001
Male	1066 (39.38)	6 (0.56)	62 (5.82)	299 (28.05)	61 (57.41)	87 (8.16)	
Female	1641 (60.62)	10 (0.61)	185 (11.27)	610 (37.17)	773 (47.11)	63 (3.84)	
Age ^a^							< 0.001
50–54	889 (32.84)	5 (0.56)	58 (6.52)	273 (30.71)	491 (55.23)	62 (6.97)	
55–59	783 (28.93)	7 (0.89)	61 (7.79)	278 (35.50)	384 (49.04)	53 (6.77)	
≥ 60	1035 (38.23)	4 (0.39)	128 (12.37)	358 (34.58)	510 (49.28)	35 (3.38)	
*Socioeconomic status* Education ^a^							< 0.001
No formal education	1702 (62.87)	14 (0.82)	190 (11.16)	615 (36.13)	807 (47.41)	76 (4.47)	
Elementary school	809 (29.89)	2 (0.25)	46 (5.69)	237 (29.30)	467 (57.73)	57 (7.05)	
Middle school and above	196 (7.24)	0 (0)	11 (5.61)	57 (29.08)	111 (56.63)	17 (8.67)	
Household annual income ^a^							< 0.010
≤ 12,000 CNY	680 (25.12)	6 (0.88)	83 (12.21)	250 (36.76)	304 (44.71)	37 (5.44)	
12,000–19,999 CNY	715 (26.41)	8 (1.12)	56 (7.83)	228 (31.89)	384 (53.71)	39 (5.45)	
20,000–59,999 CNY	856 (31.62)	1 (0.12)	80 (9.35)	278 (32.48)	443 (51.75)	54 (6.31)	
≥ 60,000 CNY	456 (16.85)	1 (0.22)	28 (6.14)	153 (33.55)	254 (55.70)	20 (4.39)	
Employment ^a^							< 0.001
Employed	738 (27.26)	10 (1.36)	50 (6.78)	208 (28.18)	406 (55.01)	64 (8.67)	
Unemployed	1969 (72.74)	6 (0.30)	197 (10.01)	701 (35.60)	979 (49.72)	86 (4.37)	
Registered permanent residence ^a^							< 0.001
Rural	2253 (83.23)	13 (0.58)	219 (9.72)	726 (32.22)	1159 (51.44)	136 (6.04)	
Urban	401 (14.81)	2 (0.50)	19 (4.74)	155 (38.65)	211 (52.62)	14 (3.49)	
Unified household	53 (1.96)	1 (1.89)	9 (16.98)	28 (52.83)	15 (28.30)	0 (0)	
Medical insurance ^a^							< 0.001
MIUE	243 (8.98)	1 (0.41)	8 (3.29)	83 (34.16)	142 (58.44)	9 (3.70)	
MIUR	358 (13.22)	1 (0.28)	27 (7.54)	143 (39.94)	167 (46.65)	20 (5.59)	
NCMS	1198 (44.26)	2 (0.17)	78 (6.51)	399 (33.30)	629 (52.50)	90 (7.51)	
MIRR	848 (31.33)	12 (1.42)	133 (15.68)	263 (31.01)	410 (48.35)	30 (3.54)	
No medical insurance	60 (2.22)	0 (0)	1 (1.67)	21 (35.00)	37 (61.67)	1 (1.67)	
*Health behaviors*							
Smoking ^a^							< 0.001
Never	2113 (78.06)	15 (0.71)	208 (9.84)	750 (35.49)	1036 (49.03)	104 (4.92)	
Ever	185 (6.83)	1 (0.54)	18 (9.73)	56 (30.27)	99 (53.51)	11 (5.95)	
Current	409 (15.11)	0 (0)	21 (5.13)	103 (25.18)	250 (61.12)	35 (8.56)	
Alcohol consumption ^a^							< 0.001
No	2000 (73.88)	15 (0.75)	216 (10.80)	693 (34.65)	978 (48.90)	98 (4.90)	
Occasionally (less than once a week)	469 (17.33)	1 (0.21)	25 (5.33)	157 (33.48)	254 (54.16)	32 (6.82)	
Frequently (more than once a week)	238 (8.79)	0 (0)	6 (2.52)	59 (24.79)	153 (64.29)	20 (8.40)	
Physical activity ^a^							> 0.050
Low (≤ 18.9 MET/day)	1353 (49.98)	14 (1.03)	123 (9.09)	476 (35.18)	656 (48.48)	84 (6.21)	
High (> 18.9 MET/day)	1354 (50.02)	2 (0.15)	124 (9.16)	433 (31.98)	729 (53.84)	66 (4.87)	
Sleep duration (per night) ^a^							< 0.010
< 7 h	184 (6.80)	4 (2.17)	27 (14.67)	65 (35.33)	81 (44.02)	7 (3.80)	
7 h–8 h	1783 (65.87)	6 (0.34)	149 (5.50)	592 (33.20)	926 (51.93)	110 (6.17)	
> 8 h	740 (27.34)	6 (0.81)	71 (9.59)	252 (34.05)	378 (51.08)	33 (4.46)	
Quality of nighttime sleep ^b^	0 (0, 1.0)	1.5 (0, 2.0)	1.0 (0, 2.0)	0 (0, 2.0)	0 (0, 1.0)	0 (0, 1.0)	< 0.001
*Chronic diseases*							
Obesity ^a^							> 0.050
No	2167 (80.05)	14 (0.65)	207 (9.55)	717 (33.09)	1104 (50.95)	125 (5.77)	
Yes	540 (19.95)	2 (0.37)	40 (7.41)	192 (35.56)	281 (52.04)	25 (4.63)	
Hypertension ^a^							< 0.001
No	1860 (68.71)	9 (0.48)	134 (7.20)	590 (31.72)	1006 (54.09)	121 (6.51)	
Yes	847 (31.29)	7 (0.83)	113 (13.34)	319 (37.66)	379 (44.75)	29 (3.42)	
Diabetes ^a^							< 0.010
No	2610 (96.42)	14 (0.54)	234 (8.96)	867 (33.22)	1353 (51.84)	142 (5.44)	
Yes	97 (3.58)	2 (2.06)	13 (13.40)	42 (43.30)	32 (32.99)	8 (8.25)	
Hyperlipidemia ^a^							< 0.001
No	2014 (74.40)	8 (0.40)	154 (7.65)	653 (32.42)	1083 (53.77)	116 (5.76)	
Yes	693 (25.60)	8 (1.15)	93 (13.42)	256 (36.94)	302 (43.58)	34 (4.91)	
*Mental health*							
Anxiety ^b^	0 (0, 1.0)	1.0 (0, 2.0)	1.0 (0, 2.0)	1.0 (0, 2.0)	0 (0, 1.0)	0 (0, 0)	< 0.001
Depression ^b^	0 (0, 1.0)	2.0 (0, 2.0)	2.0 (0, 2.0)	0 (0, 2.0)	0 (0, 1.0)	0 (0, 0)	< 0.001

^a^ n (%), ^b^ median with IQR

### Studied variables associated with self-rated health

Among all participants, 5.54% rated their health excellent, 51.16% very good, 33.58% good, 9.12% fairly poor and 0.59% poor. Sex, age, marital status, education, household annual income, employment, registered permanent residence, medical insurance, smoking, alcohol consumption, sleep duration, quality of nighttime sleep, hypertension, hyperlipidemia, obesity, and diabetes were associated with SRH (Table 1).

### Effect of studied variables on self-rated health

**Fig. 1:**
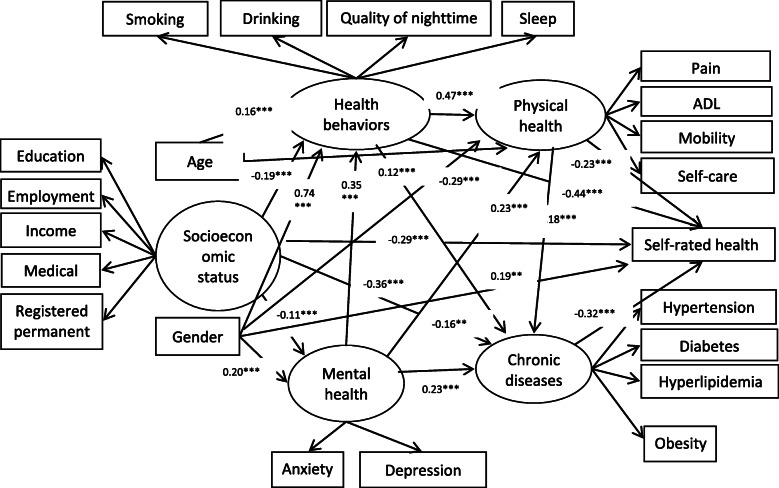
Structural equation model of SRH among Tibetan residents. Note: Values indicate standardized coefficients, ** *P* < 0.01, *** *P* < 0.001. The final structural model fit well with the data (*χ*^2^/df = 4.512, NFI = 0.919, GFI = 0.973, CFI = 0.935, IFI =0.935, TLI = 0.919, RMSEA = 0.036 and RMR = 0.041). The squared multiple correlation (R^2^) was 0.33, which indicates that the model explained 33% of the variance in SRH

The final structural model (Fig. 1) described the upstream associations of demographics, socioeconomic status, health behaviors, physical health, mental health and chronic diseases on SRH as well as their interactions, with adequate model fit.

Based on standardized path weight coefficients (β’s), physical health (*β* = − 0.23, *P* <  0.001), health behaviors (*β* = − 0.44, *P* <  0.001), socioeconomic status (*β* = − 0.29, *P* <  0.001), chronic diseases (*β* = − 0.32, *P* <  0.001) and gender (*β* = 0.19, *P* <  0.001) were directly associated with SRH among older Tibetan people. The SRH decreased by 0.23, 0.44, 0.29 and 0.32 units for every unit change in physical health, health behaviors, socioeconomic status and chronic diseases. The SRH of men was 0.19 units higher than that of women. This finding indicates that participants with higher scores on physical health reported poor SRH, and participants who had been diagnosed with hypertension, diabetes, hyperlipidemia or obesity reported poor SRH. Women seem to be more likely to rate their health as poorer than men.

Socioeconomic status exhibited a modest association with SRH. This finding indicates that lower educational attainment, unemployment, lower household annual income, and lower levels of medical insurance entitlement were risk factors for SRH.

Health behaviors were one of the strongest factors associated with SRH. This finding indicated that participants who do not drink alcohol perceived poorer health than those who drink occasionally or frequently. Nonsmokers reported poorer SRH than smokers or current smokers. High scores in the quality of nighttime sleep and short sleep duration were negatively associated with SRH.

### Estimates of direct and indirect effects

The mediating effect was tested with bias-corrected bootstrapping. The method of repeated random sampling was used to extract 2000 samples from the original data, and an approximate sampling distribution was generated. The confidence interval of the mediating effect of 95% was estimated by the 2.5 percentile and 97.5 percentile.

Table 2 indicates the total effect of all latent variables on SRH. Overall, health behavior had the greatest total effect on SRH followed by chronic diseases and mental health with standardized regression coefficients of − 0.522, − 0.323 and − 0.320, respectively. Socioeconomic status, physical health and gender affected SRH both directly and indirectly; however, the total effect of socioeconomic characteristics on SRH was not statistically significant. The direct effect of physical health on SRH was much greater than its indirect effect. The likelihood of physical health influencing SRH via mediators (such as chronic diseases) was minimal given that its indirect effect was close to zero. The indirect effect of gender on SRH was greater than its direct effect. There are potential complete mediator effects in which age and mental health affect SRH through mediators, such as physical health, health behaviors and chronic diseases.

**Table 2:** Direct and indirect effects of variables on self-rated health (SRH)

Latent Variable	Direct Effect	Indirect Effect	Total Effect
Socioeconomic status	−0.294^***^	0.252^***^	−0.042
Health behaviors	−0.439^***^	−0.083	−0.522 ^**^
Physical health	−0.230^***^	−0.059^***^	−0.289 ^***^
Mental health	0	−0.320^***^	−0.320 ^***^
Chronic diseases	− 0.323^***^	0	− 0.323^***^
Gender	0.194^*^	−0.366^***^	−0.172^***^
Age	0	−0.119^***^	−0.119^***^

* *p* < 0.05, ** *p* < 0.01, *** *p* < 0.001

## Discussion

This study used SEM to systematically examine the factors associated with SRH. By including multiple variables into one conceptual model and analyzing them simultaneously, the study is unique in its ability to describe a complex network. All five latent variables based on the conceptual framework for determining health (i.e.*,* socioeconomic status, health behaviors, physical health, mental health, and chronic diseases) were predictors of SRH.

The findings of this study demonstrate the association between socioeconomic status and SRH. First, previous studies have identified lower levels of education, lower income, and unemployment as important risk factors for SRH [43, 44], and the same conclusion was echoed in our study. Second, we found that lower levels of medical insurance entitlement were risk factors for SRH, which is similar to findings from other studies [45, 46]. A lack of medical insurance limits access to preventive services [47]. It is important to acknowledge that health insurance interventions have the potential to improve health outcomes, such as mental health and SRH [48]. In addition, China’s social health insurance schemes are linked to registered permanent residence and occupation. The medical insurance for urban residents and formal employees has better social security benefits. The link between medical insurance and SRH partially reflects occupational and urban-rural disparities. Third, this study found that registered permanent residence status is the most important socioeconomic factor affecting SRH. Under the background of the urban-rural dual structure in China [49], registered permanent residence reflects the socioeconomic status of residents. Registered permanent residence status has been widely accepted as a factor associated with many diseases [50], which obviously affects SRH [51].

Our study assessed the relationship between SRH and four health behaviors (smoking, alcohol consumption, sleep duration, and quality of nighttime sleep) simultaneously. The importance of health behaviors on individual health has been widely accepted [52]. Consistent with previous research [53], the findings of this study confirmed that poor quality of nighttime sleep and short sleep duration were negatively associated with SRH. This finding highlights the value of healthy sleep hygiene in older people. In addition, we found that drinking seemed to be a protective factor for SRH. This result is consistent with findings of the English Longitudinal Study of Aging, which reported that nondrinkers reported a significantly increased rate of self-rated poor health than those who drank occasionally [54]. However, another study conducted in the US revealed a negative association between alcohol consumption and SRH [14]. The difference with respect to this study may be related to the unique customs of the minorities. For smoking behavior, as noted in previous studies, the prevalence of poor SRH was highest among nonsmokers [55, 56]. Our results differ from previous findings, indicating that nonsmokers reported poorer SRH than smokers or current smokers. Various studies demonstrated that smokers are less concerned about the future consequences of their health behaviors than nonsmokers [57]. Our participants were mainly older people with low education levels who may have a worse understanding of the dangers of smoking. Health behaviors not only had a large direct effect but also served as an important bridge to strengthen the effect of socioeconomic status and mental health on SRH. These findings suggest that the associations between drinking, alcohol consumption and SRH may show different directions due to the influence of cultural factors and education level. Physical activity had no significant effect on SRH. It is well documented that physical activity has an important impact on health outcomes [58]. In this study, a possible explanation for the nonsignificant effect may be that participants generally reported lower levels of physical activity (18.9 MET per day), which is substantially lower than a previous study on older people in Chinese nonethnic minority areas [59].

We found that higher scores of physical health were associated with poorer SRH. Given that SRH is based on people’s subjective feelings of their physical health information [60], older people with poor physical health may be more likely to report poor SRH. We found that participants who had been diagnosed with chronic diseases were more likely to rate their health as poor, which is consistent with previous findings [2, 51, 61]. Prevention interventions for chronic diseases should be enhanced at the community level, and standardized management of patients with chronic diseases should be strengthened to improve overall health.

Interestingly, some direct and indirect associations were noted among components of the model based on the conceptual framework for determinants of health, which are worthy of discussion. First, we found that the effects of physical health on SRH involved both direct and indirect effects, and physical health played an intermediary role in the associations among health behaviors, mental health and SRH. The importance of physical health in our SEM model suggests that the health status of older people should be considered. Second, this study found that mental health had no direct effect on SRH, but it had a strong indirect effect. Many studies have demonstrated the association between mental health and SRH [62, 63]. Mental health was associated with socioeconomic status, health behaviors, physical health, chronic diseases, and gender in the model and can indirectly affect SRH through variables, such as healthy behaviors, physical health, and chronic diseases. Finally, our results confirmed that gender had a strong association with SRH, and gender was associated with mediating variables interacting with health behavior and health outcome variables in the model. Future research on SRH should not ignore the influence of gender differences.

The strength of this study is the simultaneous assessment of the association between many factors and SRH and the description of the complex interaction. These effects of factors can then be ranked according to their strength of association to identify priority areas for intervention. In addition, our participants were all Tibetans from a plateau area in China whose lifestyles and health conditions may be unique to other Chinese populations, yet remarkably little is known about these topics. We hope that the information can guide the local health intervention, such as prevention and control of chronic diseases.

There were several limitations in this study. First, the data we used were from a cross-sectional study and thus cannot allow one to infer causality. Second, the use of self-report data for some variables can introduce recall bias. For instance, sleep duration was self-reported, so it may not be exactly accurate. Third, the model presented in this study represents only a fraction of the overall impacts of SRH. For example, social networks demonstrate a unique positive association with SRH [64], and this was not assessed in this study given that no data on social networks were collected.

## Conclusion

Based on the conceptual framework for determinants of health, this study first described the complex network of factors concomitantly associated with self-rated health (SRH) among the older population in a Chinese plateau area. Our findings suggested that interventions targeting behavioral changes, health and chronic disease management should be attached to improve SRH among older populations in the plateau area without ignoring gender differences and socioeconomic disparities. More studies of variables affecting SRH interactions based on the conceptual framework for determinants of health might be needed to provide information for targeted interventions of SRH improvement among other subgroups.

## Data Availability

The datasets used and analysed during the current study are available from the corresponding author on reasonable request.
